# Medication adherence and glycemic control among diabetes patients in developing countries

**DOI:** 10.1186/s41043-019-0198-9

**Published:** 2019-11-12

**Authors:** Saurav Basu

**Affiliations:** Department of Community Medicine, Maulana Azad Medical College, Bahadur Shah Zafar Marg, New Delhi, 110002 India

**Keywords:** Diabetes, Adherence, Glycemic control, Developing countries

## Abstract

The potential interconnectedness of medication adherence, glycemic control, and clinical inertia in resource-constrained settings of the developing world needs further evaluation.

Dear Editor,

Low- and middle-income countries account for 80% of the global diabetes burden [1]. Moreover, it is well established that suboptimal medication adherence in diabetes patients due to lack of timely refill replenishment arising from health system and socioeconomic factors is a major public health challenge in developing countries [2].

The study by Rathish et al. from a rural province in Sri Lanka makes an important contribution to the sparse literature on the subject in the country and establishes the benefits of government-funded universal free-health services in enhancing medication coverage and adherence in low-income diabetes patients [3]. However, there are certain concerns regarding the study methodology and interpretation that are discussed below.

The researchers applied the Morisky-Green-Levine scale to assess medication adherence in diabetes patients. The scale has low sensitivity and doubtful validity in this patient population that is further lowered by the application of a cutoff score of 2 that indicates moderate adherence only which might be inadequate in the attainment of good glycemic control. The direct comparison of the rates of adherence of this study with other studies that applied a different medication adherence scale and/or a different adherence definition is inappropriate as more sensitive scales and a more stringent definition of non-adherence could yield different adherence rates.

A major limitation of the present study is also the lack of reporting of glycemic control status in the patients. This is particularly important for the identification of the phenomenon of clinical or therapeutic inertia indicating the failure to adequately intensify the treatment in patients showing poor glycemic control that increases the risk of incident diabetes-related complications. Previous studies from government tertiary care hospitals in India that provide free medication coverage have observed a high prevalence of poor glycemic control despite good anti-diabetes medication adherence rates in the patients [4, 5]. This has been attributed to clinical inertia from limited availability of anti-diabetic drug armamentarium and failure of a timely switch to insulin therapy [6, 7]. A study in Brazil also observed a high-rate of clinical inertia during diabetes care [8]. The Rathish et al. [3] study had reported the lack of availability of some anti-diabetes agents like DPP-4 inhibitors in the free service compared to the paid service which could have significant implications on patient glycemic status. Future studies from the region should therefore evaluate the potential interconnectedness of adherence, glycemic control, and clinical inertia in their settings.

## Data Availability

Not applicable
